# How to survive a nerve-wracking journey

**DOI:** 10.7554/eLife.01845

**Published:** 2013-12-17

**Authors:** Andrea Yung, Lisa V Goodrich

**Affiliations:** 1 **Andrea Yung** is in the Department of Neurobiology, Harvard Medical School, Boston, United Statesandreayung@fas.harvard.edu; 2 **Lisa V Goodrich** is in the Department of Neurobiology, Harvard Medical School, Boston, United Stateslisa_goodrich@hms.harvard.edu

**Keywords:** planar cell polarity (PCP), axon growth, limb innervation, neural crest, cell death, muscle atrophy, Mouse

## Abstract

When the axons that carry signals to muscles are growing, they rely on help from Frizzled3—a protein that is known to perform a number of other important functions in cells—to reach their final destination.

**Related research article** Hua ZL, Smallwood PM, Nathans J. 2013. *Frizzled3* controls axonal development in distinct populations of cranial and spinal motor neurons. *eLife* **2**:e01482. doi: 10.7554/eLife.01482

**Image** Dorsally projecting motor axons in mutant mice

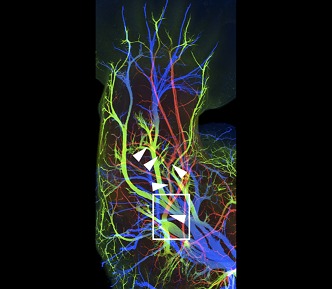

Although many nerve fibres travel considerable distances as they develop, the motor axons that carry signals from the central nervous system to various muscles throughout the body must complete the neuronal equivalent of the Oregon Trail. Remarkably, these motor axons usually reach their targets and understanding how this happens has motivated generations of developmental neurobiologists.

The discovery of axon guidance molecules in the 1990s represented a major breakthrough in our efforts to understand how axons manoeuvre through the body to reach their final targets (Evans and Bashaw, 2010). Just as landmarks helped the early pioneers navigate their way along the Oregon trail to the western frontier of the US, guidance molecules provide axons with directions to their destination. Unfortunately, as with the pioneers, the survival of the neurons often depends on their axons making it to their final destination.

It has been hypothesized that intermediate targets might also provide such support en route, which has the effect of eliminating axons that stray off the trail early on (Wang and Tessier-Lavigne, 1999). However, in vivo evidence for this model has remained scarce. Now, in *eLife*, Zhong Hua, Philip Smallwood and Jeremy Nathans of the Johns Hopkins University School of Medicine present new evidence linking the arrival of motor axons at intermediate targets with neuronal survival (Hua et al., 2013).

Muscles in the limbs receive input from neurons in the lateral motor column of the spinal cord. These neurons are further divided into lateral and medial groups, and although the axons projecting from these neurons leave the spinal cord together, their paths diverge within the limb to target dorsal and ventral muscles respectively (Figure 1A). Axons at this branching point must decide which region of the limb to enter, and then continue growing to reach all the muscles that control the movement of that limb. So how do the axons decide which road to take? The answer lies in the combinations of axon guidance signals provided by the developing limb, which give specific directions about where each axon should and should not grow (Bonanomi and Pfaff, 2010). In the absence of these signals, a dorsal axon might accidentally travel to the ventral muscles, or vice versa, resulting in what is called a guidance phenotype (Figure 1B; Helmbacher et al., 2000; Huber et al., 2005; Luria et al., 2008).

**Figure 1.**
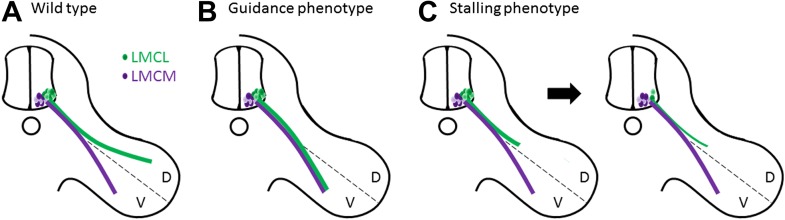
Motor axons usually reach their target, but sometimes they lose their way or stop growing and die. (**A**) Two groups of neurons in the lateral motor column of the spinal cord target the developing limb. Although they leave the spinal cord together, their paths later diverge, with the neurons in the lateral group (green) innervating the dorsal (D) musculature and the neurons in the medial group (purple) innervating the ventral (V) musculature. Guidance molecules ensure that both sets of neurons reach the correct target. (**B**) In the absence of certain guidance molecules, one set of neurons might arrive at the wrong target. This defect is known as a guidance phenotype. (**C**) Hua et al. have discovered a pure stalling phenotype in which motor axons belonging to neurons in the lateral group set out on the correct path but stop growing, which results in the death of these neurons in the spinal cord.

When an axon encounters a guidance signal, it must choose to continue growing straight ahead, make a turn, or perhaps stop altogether. The decision is made within a specialized structure at the tip of the growing axon called the growth cone. How the growth cone actually responds to axon guidance signals remains poorly understood, but recent research into planar polarity proteins has unexpectedly uncovered a possible mechanism. 

Planar polarity proteins help ensure that cells are properly aligned with their surrounding tissue (Wang and Nathans, 2007). As would be expected, the absence of planar polarity proteins impairs the formation of tissues that rely on cell alignment, such as hairs on the surface of our skin. However, the absence of Frizzled3 (Fz3)—a planar polarity protein that acts as a receptor in the well-known Wnt signalling pathway—also leads to serious defects in the central nervous system, notably a complete absence of many nerve tracts (Wang et al., 2002). These phenotypes seem to be caused by changes in the ability of growth cones to turn towards Wnt signals (Lyuksyutova et al., 2003; Shafer et al., 2011), confirming that planar polarity proteins ‘give directions’ to the axons, just like axon guidance molecules do.

In the case of motor neurons, Hua et al. find an unusual type of defect in *Fz3* mutant mice. Through a detailed analysis of innervation patterns in the limbs, they show that the absence of Fz3 causes axons that should travel to the dorsal muscles to instead stall at the branch point (Figure 1C). Thus, these axons follow the right path but stop growing, likely because their growth cones cannot figure out which way to go. This stalling phenotype is fundamentally different from previously described guidance phenotypes, where the axons do eventually reach a target, albeit the wrong one.

Moreover, the stalling defect has a surprising consequence. When an axon fails to reach its final destination, the neuron in the lateral motor column to which it belongs actually dies. However, these neurons die two days earlier than expected. This suggests that they die because they do not receive support from an intermediate target: in other words, the axons never make it to the gas station to re-fuel.

Though axon stalling has been observed in other mouse mutants, this is a rare example where pure stalling is tied to cell death, providing important in vivo evidence that axons receive intermediate survival cues during their journey. Intriguingly, other cranial nerves also stall in *Fz3* mutants, yet only a subset show an increase in cell death, suggesting that not all nerves require intermediate support. Are certain motor axons predisposed to require such support? Or do the unaffected motor axons rely on other members of the Frizzled family for their survival? What are the survival signals themselves, and do the same proteins also help axons choose their route?

It will also be important to determine how the failure of motor axons to extend fully into the limb fits with Fz3’s known role in guidance: are the nerves stalled because they don’t know which way to turn, or could this be an effect on the ability of the axon to grow at all? Understanding how all these pathways coalesce to produce a stereotyped trail of axon growth is proving to be an exciting path of research—thankfully, it does not involve the same perils.
